# Magnetic Solid-Phase Microextraction Protocol Based on Didodecyldimethylammonium Bromide-Functionalized Nanoparticles for the Quantification of Epirubicin in Biological Matrices

**DOI:** 10.3390/pharmaceutics15041227

**Published:** 2023-04-12

**Authors:** Natalia Treder, Natalia Szuszczewicz, Anna Roszkowska, Ilona Olędzka, Tomasz Bączek, Ewa Bień, Małgorzata Anna Krawczyk, Alina Plenis

**Affiliations:** 1 Department of Analytical Chemistry, Medical University of Gdansk, 80-416 Gdansk, Poland; 2 Department of Pharmaceutical Chemistry, Medical University of Gdansk, 80-416 Gdansk, Poland; 3 Department of Pediatrics, Hematology and Oncology, Medical University Gdansk, 80-211 Gdansk, Poland

**Keywords:** epirubicin, magnetic solid-phase microextraction, nanoparticles, validation, drug monitoring

## Abstract

Due to epirubicin’s (EPI) narrow therapeutic index and risk of cardiotoxicity, it is critical to monitor concentrations of this drug when being used to treat cancer patients. In this study, a simple and fast magnetic solid-phase microextraction (MSPME) protocol for the determination of EPI in plasma and urine samples is developed and tested. Experiments were performed using prepared Fe_3_O_4_-based nanoparticles coated with silica and a double-chain surfactant—namely, didodecyldimethylammonium bromide (DDAB)—as a magnetic sorbent. All the prepared samples were analyzed via liquid chromatography coupled with fluorescence detection (LC-FL). The validation parameters indicated good linearity in the range of 0.001–1 µg/mL with a correlation coefficient > 0.9996 for plasma samples, and in the range of 0.001–10 µg/mL with a correlation coefficient > 0.9997 for urine samples. The limit of detection (LOD) and limit of quantification (LOQ) for both matrices were estimated at 0.0005 µg/mL and 0.001 µg/mL, respectively. The analyte recovery after sample pretreatment was 80 ± 5% for the plasma samples and 90 ± 3% for the urine samples. The developed method’s applicability for monitoring EPI concentrations was evaluated by employing it to analyze real plasma and urine samples collected from a pediatric cancer patient. The obtained results confirmed the proposed MSPME-based method’s usefulness, and enabled the determination of the EPI concentration–time profile in the studied patient. The miniaturization of the sampling procedure, along with the significant reduction in pre-treatment steps, make the proposed protocol a promising alternative to routine approaches to monitoring EPI levels in clinical laboratories.

## 1. Introduction

Over the last 30 years, epirubicin (EPI)—a DNA topoisomerase II enzyme-targeted cytostatic drug and one of the most common anthracycline antibiotics—has been shown to be effective in chemotherapy regimens for breast cancer, ovarian cancer, and lymphomas, among others [1,2,3]. Given this success, researchers have continued to conduct clinical trials exploring the efficacy of EPI in treatments for patients with colorectal cancer or extramammary Paget’s disease [4,5]. However, due to EPI’s narrow therapeutic index, achieving good therapeutic results, regardless of the cancer type, depends on determining the correct drug dosage, as exceeding EPI’s therapeutic range may lead to life-threatening side effects, especially cardiotoxicity [6]. The standard dosage of EPI is based on body surface area (BSA), but this approach can result in ineffective therapies or even adverse outcomes due to inter- and intraindividual pharmacokinetic differences between patients. To overcome these limitations, specialists in various fields are working to develop new forms of EPI that target cancer cells more effectively, and they are also striving to gain a better understanding of the underlying genetic mechanisms responsible for drug-induced cardiotoxicity [7]. Nonetheless, despite obtaining better insight into these underlying mechanisms, monitoring the patient’s EPI levels and adjusting the dose accordingly remains the best approach.

Current therapeutic drug monitoring (TDM) approaches involve the determination of drug concentrations via immunoassay tests or chromatographic techniques. Immunoassay tests are advantageous for their simplicity and speed of analysis, but they are also considered less precise and reliable compared to chromatographic techniques. Despite clinical recommendations, the oncological application of TDM is limited to three cytostatic drugs (i.e., busulfan, methotrexate, and carboplatin [8]), with EPI monitoring still being highly underutilized in routine clinical practice, despite frequently being prescribed as part of oncological treatment regimens. Literature data indicated that previously proposed EPI protocols were based on liquid chromatography (LC) techniques combined with solid-phase extraction (SPE) [9,10] or liquid–liquid extraction (LLE) [6]. However, although SPE and LLE are well-studied and widely used extraction methods, they possess several significant disadvantages, including multi-step, complex, and time-consuming extraction procedures and remain a challenge for modern laboratories, consequently limiting the application of TDM in clinical practice [11].

The search for new sample-preparation solutions capable of overcoming the drawbacks of traditional techniques has led to the development of new extraction techniques in recent years. One such technique is magnetic solid-phase extraction (MSPE), which is based on the use of coated magnetic cores as magnetic sorbents that are added to a sample in amounts comparable to those used in SPE cartridges (≥30 mg) [12]. The application of MSPE has been reported for the separation of analytes from various types of matrices, including biological, environmental, and food samples. For instance, Heidari et al. used magnetic nanoparticles (MNPs) to isolate four pharmaceuticals from plasma samples without the need for a deproteinization step, obtaining a simple baseline in chromatograms for biological matrices [13]. The advantages of this alternative SPE method were also highlighted by Zhang et al., who used magnetic sorbents to separate antiepileptic drugs from urine and plasma samples, which allowed them to obtain similar results to traditional extraction methods, only with shorter sorption and overall analysis times [14]. Despite the progress made in SPE-based sample preparation, the selection of the nanoparticle (NP) coating remains an essential and common issue with MSPE. The use of surfactants is an interesting approach in the functionalization of NPs, as these chemicals possess hydrophobic and hydrophilic portions, as well as different orientations, on their surface, which allows the sorbents to be adjusted based on the experimental conditions. Due to the electrostatic and hydrophobic interactions of surfactants, the use of these positively or negatively charged structures with long hydrophobic chains can improve the adsorption of analytes onto the sorbent surface, thus providing higher extraction efficiencies compared to uncoated NPs [15,16].

In the present study, a simple and fast magnetic solid-phase microextraction (MSPME) method is employed to extract EPI from urine and plasma samples, followed by analysis via LC-FL. The proposed microsampling protocol is based on the use of MNPs coated with didodecyldimethylammonium bromide (DDAB) (Figure S1), which are prepared according to a procedure described in our previous work [17]. In the first stage of experiments, we examine the impact of variables such as the amounts of NP and coating material, pH, extraction and desorption times, and the effect of salting on the extraction of EPI from a biological matrix. The obtained validation parameters confirmed that the optimized method satisfies the current requirements of the International Council for Harmonization of Technical Requirements for Pharmaceuticals for Human Use (ICH) [18] and the Food and Drug Administration (FDA) [19]. Ultimately, the proposed MSPME-LC-FL method is applied for the analysis of EPI in real urine and plasma samples collected from pediatric cancer patients during two cycles of chemotherapy, with the resultant data being used to determine the concentration–time profiles of EPI in both matrices. Aside from providing greater insight into how the double-chain surfactant used in NP functionalization influences the method’s extraction efficiency for EPI, this study is the first to report a chromatographic method for the determination of EPI in real human plasma and urine samples that is based on a microextraction procedure.

## 2. Materials and Methods

### 2.1. The Chemicals and Reagents

Iron (III) chloride hexahydrate (FeCl_3_·6H_2_O) and iron (II) sulfate heptahydrate (FeSO_4_·7H_2_O) were supplied by Chempur (Piekary Śląskie, Poland). Tetraethyl orthosilicate (TEOS), formic acid, methanol (MeOH), and HPLC-grade acetonitrile (ACN) were purchased from Merck (Poznań, Poland), while sodium hydroxide (NaOH), ammonium hydroxide solution (NH_4_OH, 25%), ethanol (EtOH, 96% Pure P.A.), hydrochloric acid (HCl) at a concentration of 36.6%, and acetone were purchased from POCH (Gliwice, Poland). Didodecyldimethylammonium bromide (DDAB) (Figure S1) was obtained from Sigma-Aldrich (St. Louis, MO, USA), and epirubicin hydrochloride (EPI) and daunorubicin hydrochloride (DAU), which was used as internal standard (IS) (>98% purity), were purchased from Cayman Chemical Company (Ann Arbor, MI, USA). The water used in the experiments was deionized using a Millipore (Molsheim, France) Milli-Q water purification system. The control artificial human plasma (citrated plasma, cat. no. P9523-5ML) was provided by Merck (Poznań, Poland). The 1.5 M NaOH solution was prepared by diluting the appropriate amounts of solid salt in ultrapure water. The 0.1 M HCl was prepared by diluting the appropriate amounts of the concentrated acid in ultrapure water.

### 2.2. Chromatographic Conditions

Chromatographic analysis was performed on an Agilent Technologies 1220 Infinity LC system equipped with a gradient pump, an autosampler, and a thermostat (Agilent 1200 G4290C, Agilent Technologies, Santa Clara, CA, USA) that had been combined with a fluorescence detector (LC-FL) (Shimadzu RF-20A XS, Kyoto, Japan). The separation of EPI and the IS was achieved on a Synergi Hydro-RP 80A column (150 × 4.5 mm, 4 µm; Phenomenex, Torrance, CA, USA). The mobile phases consisted of 0.1% aqueous solution formic acid (component A) and ACN (component B). The following gradient elution program was employed: 0–2 min, 5–30% B; 2–8 min, 30–50% B; 8–10 min, 50–95% B; 10–10.1 min, 95–5% B; and 10.1–16 min, re-equilibration time. The monitoring wavelengths were set at 487 nm and 547 nm for excitation and emissions, respectively. All analyses were performed using a flow rate of 1.0 mL/min, an injection volume of 5 μL, and a column oven temperature of 30 °C. Agilent ChemStation software A.01.04 was used for instrument control, data acquisition, and processing.

### 2.3. Stock and Working Standard Solutions

Stock standard solutions of EPI and IS were prepared in MeOH at a concentration of 1 mg/mL. Working standard solutions of EPI at concentrations of 500, 100, 50, 10, 5, 1, 0.1, and 0.05 µg/mL and IS at concentrations of 50 or 25 µg/mL were prepared by diluting the stock standard solution and subsequent working solutions. All obtained solutions were stored at −80 °C until further use.

### 2.4. Calibration and Quality Control Samples

Calibration samples ranging between 0.001 and 1 µg/mL for plasma and 0.1 and 10 µg/mL for urine were prepared according to the same MSPME procedure. Briefly, the appropriate volume of EPI working standard solution was added to 0.5 mL of blank biological sample placed in Eppendorf tubes (2 mL). Samples were also spiked with IS working standard solution to obtain final concentrations of 0.5 µg/mL and 1.0 µg/mL for plasma and urine, respectively. The prepared samples were then added to the Eppendorf tubes along with the magnetic sorbents (details relating to the preparation of the magnetic sorbent are presented in Appendix A). After extraction, the MNPs were separated from the solution using an external magnetic field, and the solution was decanted. To obtain lower LOQ parameters, 50 µL and 100 µL of ACN were added to calibration plasma and urine samples spiked with EPI at 0.001 µg/mL and higher concentration, respectively. The mixture was stirred for 5 min at 3000 rpm, separated from the magnetic sorbents again using an external magnetic field, and then finally transferred to the inserts for analysis. All samples spiked with EPI or IS at concentrations above 1 µg/mL were dissolved in 200 µL of ACN to prevent fluorescence quenching in the chromatograms and to provide accurate analyte detection.

Quality control samples (QCs) were prepared at 0.1, 0.5, and 1 µg/mL for plasma and 0.5, 3, and 7.5 µg/mL for urine samples by adding the appropriate volume of EPI working standard solution to obtain the desired low (LQC), medium (MQC), and high (HQC) concentration levels. All plasma and urine QCs were spiked with IS at 0.5 µg/mL and 1 µg/mL, respectively, and prepared in triplicate according to the extraction protocol for calibration samples described above.

### 2.5. Method Validation

Validation parameters including linearity, limits of detection (LOD), limits of quantification (LOQ), selectivity, precision, accuracy, stability and recovery were calculated in accordance with FDA and ICH guidelines [18,19].

The method’s linearity was determined by analyzing plasma calibration samples with EPI concentrations of 0.001, 0.05, 0.1, 0.25, 0.5, and 0.75, 1 µg/mL and an IS concentration of 0.5 µg/mL, and urine samples with EPI concentrations of 0.001, 0.1, 0.5, 1, 3, 5, 7.5, and 10 µg/mL and an IS concentration of 1.0 µg/mL. The linear calibration curves were constructed by plotting the measured ratios of the peak areas of EPI and IS versus the concentrations of analyte that were spiked to the samples (n = 6). The LOD and LOQ values were calculated using signal-to noise ratios (S/N) of 3 (n = 6) and 10, respectively, which resulted in an LOQ with precision of <15% and accuracy of between 80–120% (n = 6). The method’s selectivity was verified by analyzing unspiked plasma and urine samples, as well as plasma and urine samples spiked with EPI at concentrations of 0.1 µg/mL and 0.5 µg/mL, respectively, and IS at concentrations of 0.5 µg/mL and 1 µg/mL, respectively. Six replicates were prepared for each of the plasma and urine QC samples containing low (LQC), medium (MQC), and high (HQC) levels of EPI and IS, followed by analysis on the same day and on separate days to validate the model’s intra- and inter-day precision and accuracy. The method’s precision was expressed as the relative standard deviation (%RSD), whereas its accuracy was defined as the percentage of relative error (%RE) calculated by comparing the mean concentration of the analyte measured via chromatographic analysis to a nominal concentration spiked to the biological material. The analyte concentration in the spiked samples was calculated as the peak area ratio of EPI to the IS (DAU). The QCs prepared with low, medium, and high levels of EPI (according to the procedure described in Section 2.4) were also used to verify the stability of the analyte and IS in urine and plasma samples during storage under the following conditions: at 4 °C in an autosampler (24 h; post-preparative stability); at room temperature (4 h; short-term thermal stability); and at −80 °C (2 months; long-term thermal stability). In addition, the analyses also included three freeze-thaw cycles to confirm the analytes’ stability under the experimental conditions. The interval for the acceptance of sample stability was set at 85–115% of analyte and IS recovery. The absolute recoveries of EPI and IS from both matrices were estimated based on the analysis of samples spiked with these compounds before and after the extraction procedure. The final concentrations of EPI in the analyzed samples were set at 0.05 and 0.25 µg/mL for plasma and 0.1 and 1 µg/mL for the urine samples. The IS concentration was adjusted to the matrices and was equal to 0.5 µg/mL for plasma samples and 1.0 µg/mL for urine samples.

### 2.6. Real Samples

The analysis of real samples was approved by the Bioethics Committee for Scientific Research at the Medical University of Gdansk (Nos. NKBBN/232/2015 and NKBBN/232-219/2021). Urine and plasma samples were collected from a nearly 12-year-old female patient in the Department of Pediatrics, Hematology and Oncology at the University Clinical Center in Gdansk, who been diagnosed with undifferentiated rhabdomyosarcoma of the liver. The patient was receiving two cycles of 1-day chemotherapy at 6-month intervals according to the following chemotherapeutic regiment: vincristine at 1.5 mg/m^2^, carboplatin at 500 mg/m^2^, and EPI at 150 mg/m^2^. The EPI was administered via intravenous (IV) infusion over a 6 h period. Additionally, during and after chemotherapy, the patient also received ondansetron (orally), dexamethasone (IV), metoclopramide (IV), and Optilyte infusion solution (IV).

Plasma samples were collected from the patient at seven time points in each chemotherapy cycle: prior to the infusion (control sample) and 2, 4, 6, 8, 12, and 24 h afterwards. The urine analysis used six samples collected from patient after the first cycle of chemotherapy. Samples were obtained before the start of EPI infusion, mid-infusion, at the end of the infusion, and at 4–8 h, 10–14 h, and 22–26 h after the end of the infusion. The urine and plasma samples were both prepared according to the extraction procedure described in Section 2.4 and were stored at −80 °C until analysis.

## 3. Results and Discussion

### 3.1. Optimization of Extraction Procedure

To achieve the highest extraction efficiency for EPI from biological matrices using the developed MSPME procedure, several experimental variables were tested, including the amounts of NP and coating material, pH values, the salting-out effect, and extraction and desorption times. These tests were performed using plasma samples spiked with EPI at 0.5 µg/mL and IS at 1.0 µg/mL, with the extraction results being evaluated based on the peak areas of both analytes.

#### 3.1.1. Amount of DDAB

In MSPME, the adsorption of the analyte mainly depends on the NPs selected as a coating material, as these particles directly determine the sorbent-analyte interactions. In our previous study [17], wherein we tested a wide range of structures for the functionalization of NPs, NPs with a double coating layer—namely, silica as the first layer and DDAB as the second layer—were identified as the most promising magnetic sorbents, as double alkyl chain surfactants provided significantly better results compared to compounds with a single chain. Moreover, in this study, the potential of DDAB in NP functionalization is exploited to develop a novel Fe_3_O_4_-based microsampling protocol. This marks the first documentation of such a procedure, as the application of surfactants has strictly been limited to single-chain structures, mainly cetyltrimethylammonium bromide (CTAB) and sodium dodecyl sulfate (SDS), in previous works [15,20,21,22].

**Figure 1:**
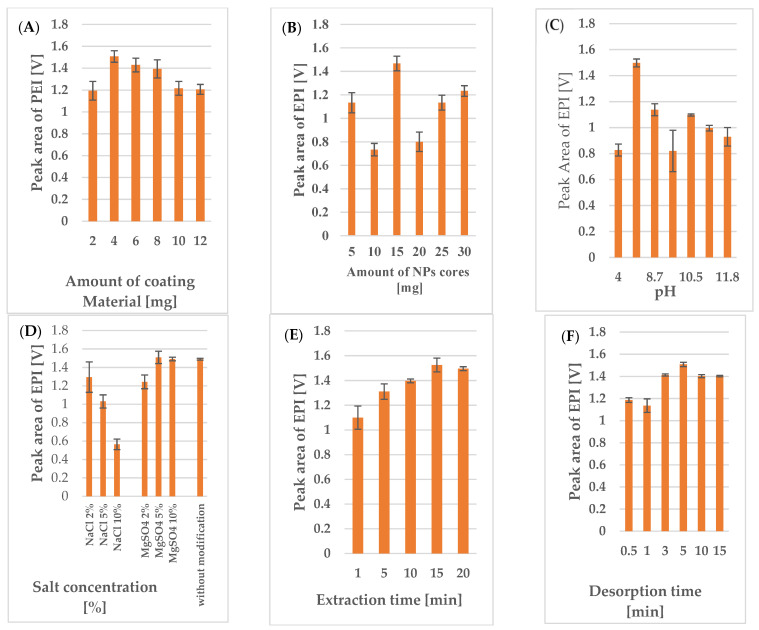
Effects of (**A**) the amount of coating material, (**B**) the amount of NP cores, (**C**) pH, (**D**) salting-out, (**E**) extraction time, and (**F**) desorption time on the peak area of EPI (0.5 µg/mL) obtained via LC-FL analysis.

The amount of DDAB affects its orientation on the NPs’ surface, and thus, the coating’s sorption capacity. To obtain the highest extraction efficiency, experiments using 15 mg magnetic cores functionalized with 2, 4, 6, 8, 10, or 12 mg of DDAB were performed. The obtained results (Figure 1A) revealed that the highest extraction efficiency was obtained using NPs coated with 4 mg of the coating material. Considering previous reports concerning the behavior of compounds with long alkyl chains (such as the NP coating materials used in the experimental conditions [23,24]), it would seem that the highest extraction results would be obtained when the DDA cation forms an irregular bilayer. The NR_4_^+^ headgroups from the first layer bind to the silica coating of the NP cores via electrostatic interactions, and the long alkyl chains of the first and second layers are bound by Van der Waals forces, while the NR_4_^+^ groups of the second layer are directed towards the hydrophilic matrix (plasma/urine samples), causing the positively charged analytes to interact with the alkyl chains of the DDA cation in the irregular bilayer due to hydrophobic and other electrostatic interactions (i.e., Van der Waals forces) [25,26,27]. Hence, the reduction in extraction efficiency observed with smaller amounts of coating material may be caused by the insufficient functionalization of the NPs. Conversely, the decreased extraction efficiency observed at higher DDAB concentrations (>4 mg) may be related to the better organization of the irregular two-layer coating, which reduces the analyte’s access to the long alkyl chains, or the tendency for the bilayer to transform into vesicles with encapsulated positively charged analytes dispersed throughout the matrix. Based on the obtained results, 4 mg of DDAB was selected as the optimal coating material amount for the subsequent optimization steps.

#### 3.1.2. Amount of Adsorbent

To determine the optimal amount of adsorbent, samples were prepared with a range of MNPs (10–30 mg) and a constant amount of coating material (i.e., 4 mg of DDAB). The obtained results (Figure 1B) indicated that the highest extraction efficiency was achieved with 15 mg of magnetic sorbents (RSD (%) = 6%), as lower adsorption was observed for ESI and the IS for the smaller (10 mg, RSD (%) = 18%) and larger amounts of sorbent (20, 25, 30 mg). This result may suggest that the smaller amounts of NPs did not provide sufficient specific surface area for analyte adsorption, while the diminished adsorption observed with the higher amounts (>15 mg) may have been related to changes in the NP coating layer (i.e., a constant amount of DDAB for different volumes of magnetic cores). These hypotheses posit that the amount of coating material should be increased to obtain a higher extraction efficiency for higher amounts of NPs (the result calculated after using 10 mg NPs can be related with higher measurement uncertainty). However, as the miniaturization of the extraction procedure remained a key issue, subsequent optimization experiments were conducted using 4 mg of DDAB and 15 mg of NP cores.

#### 3.1.3. Effect of pH

Since the extraction efficiency is dependent on the interactions between the analyte and the magnetic sorbent’s, it is critical to examine pH, as it can alter the polarity of the analyzed compounds. As such, the effects of pHs ranging from 4 to 11.7 on the extraction efficiency were tested by adding the appropriate volume of 0.1 M HCl or 0.1 M NaOH solution. The results obtained for the acidified and base samples were compared with those of a reference sample (pH 8) that had not been subjected to pH modification (Figure 1C). The obtained results confirmed that the pH value significantly influenced the extraction efficiency. However, the tested anthracycline antibiotics (EPI and DAU), which are basic compounds with a pKa ≥ 8, exhibited weaker adsorption to the sorbent surface above and below this pH value. Thus, the best extraction efficiency for these analytes took occurred in the samples that had not undergone pH modification, as the sample pH of 8 was slightly lower than the pKa of the analyte and IS.

#### 3.1.4. Salting-Out Effect

Another important factor that can affect the extraction efficiency is the salting-out effect caused by the presence of inorganic salt in the sample solution. For these experiments, we tested two salts (i.e., NaCl and MgSO_4_) at three concentration levels (2, 5 and 10%). The results presented in Figure 1D show the different effect of each salt on the extraction efficiency. As can be seen, increasing the NaCl concentration (2, 5 or 10%) or MgSO_4_ (2%) caused a significant decrease in extraction efficiency, while increasing the concentration of MgSO_4_ to 5 or 10% resulted in a corresponding increase in the adsorption of the analytes to the sorbent surface. Nevertheless, the addition of MgSO_4_ only slightly increased the method’s extraction efficiency compared to the effects observed for the sample without the addition of salt. Therefore, to minimize the impact of this variable and to simplify the procedure, subsequent experiments were performed without adding salt to the sample.

#### 3.1.5. Extraction and Desorption Time

Next, a series of experiments were conducted to optimize the extraction and desorption times. To estimate the equilibrium time enabling the maximum loading of analytes onto the sorbent, adsorptions were carried out for 1, 5, 10, 15, and 20 min, and desorption was performed for 5 min. Similarly, the equilibrium time required for maximum analyte transfer from the sorbent to the organic solvent was determined by assessing desorption times of 0.5, 1, 3, 5, 10, and 15 min, with an extraction time of 15 min. As shown in Figure 1E,F, the extraction efficiency increased up to an extraction time of 15 min and a desorption time of 5 min, and either plateaued or slightly decreased beyond this point. Thus, an extraction time of 15 min and a desorption time of 5 min were selected for use in subsequent experiments.

### 3.2. Method Validation

#### 3.2.1. Linearity

Analysis of the calibration samples (Section 2.4) confirmed the method’s linearity for both tested matrices. The correlation coefficient (*R*^2^) for the plasma samples spiked with EPI at seven calibration levels (0.001, 0.05, 0.1, 0.25, 0.5, 0.75, and 1.0 µg/mL) was 0.9996, with a slope of 1.4741 and an intercept of 0.0192. For the urine samples spiked with EPI at eight calibration levels (0.001, 0.1, 0.5, 1.0, 3.0, 5.0, 7.5, and 10.0 µg/mL), the *R*^2^ value was equal to 0.9997, with a slope of 0.6796 and an intercept of 0.0039. All results for each calibration level in the plasma and urine samples were obtained via the analysis of samples spiked at six equivalent concentrations.

#### 3.2.2. LOD and LOQ

The experimentally determined LOD and LOQ levels were 0.5 ng/mL and 1 ng/mL, respectively, for plasma and urine samples.

#### 3.2.3. Selectivity

**Figure 2:**
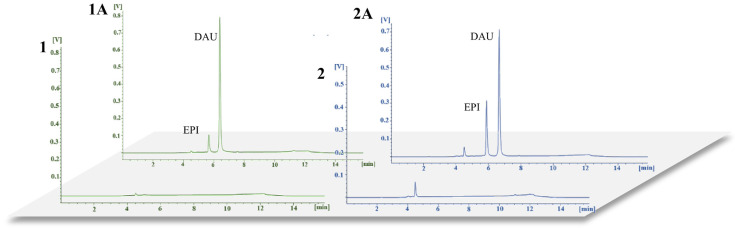
Representative chromatograms for (**1**) blank plasma samples and (**1A**) plasma samples enriched with EPI (0.1 µg/mL) and DAU (0.5 µg/mL), and for (**2**) blank urine samples and (**2A**) urine samples enriched with EPI (0.5 µg/mL) and DAU (1.0 µg/mL).

Analyses of blank plasma and urine samples and samples spiked with EPI and IS confirmed a lack of interference in the blank samples at retention times for the analyte and IS. The obtained chromatograms are shown in Figure 2.

#### 3.2.4. Inter- and Intra-Day Precision and Accuracy

The results of the QC analyses at the low, medium, and high concentration levels confirmed that the developed method’s inter- and intra-day precision and accuracy were compliant with FDA and ICH standards. Detailed data relating to the method’s precision and accuracy are presented in Table 1. As can be seen, the RSD (%) values for the intra- and inter-day precision for the plasma QCs was below 6% and 8%, respectively, and below 7% and 9%, respectively, for the urine QCs. Accuracy measured on the same and different days of analysis for the plasma QCs ranged from 98 to 103% and 95 to 102%, respectively, and from 90 to 99% and 90 to 104%, respectively, for the urine QC samples. Overall, all the precision and accuracy values calculated in this study were below the limits for analytical methods mandated by the FDA and ICH.

#### 3.2.5. Stability

A comparison of the QC analysis results for the different conditions (Section 2.5) with those of freshly prepared samples confirmed the stability of the analyzed samples. The recoveries of EPI and IS in the samples stored at −80 °C and other post-preparative storage conditions fell within the required limit of 100 ± 15% (Table S1).

#### 3.2.6. Extraction Recovery

The average extraction recoveries of EPI and IS in plasma samples spiked with standards “before” extraction compared to samples spiked with standard “after” extraction were 80 ± 5% for EPI and 82 ± 4% for the IS. Using the same measurement approach, average extraction recovery values of 90 ± 3% for EPI and 92 ± 8% for the IS were obtained for the urine samples.

### 3.3. Time–Concentration Profile of EPI in Patient Samples

In the final stage of this study, the developed method’s potential for use in clinical applications was assessed by employing it to monitor the EPI concentration profile in plasma and urine samples acquired from an 11-year-old (nearly 12 years old) female patient who was receiving a 150 mg/m^2^ dose of this drug via a 6 h infusion at as part of a 1-day chemotherapeutic regimen. A detailed description of the sampling scheme was presented in Section 2.6. Real urine and plasma samples were prepared according to the procedure detailed for the calibration and QC samples in Section 2.4, and analysis was performed under the chromatographic conditions described in Section 2.2.

**Figure 3:**
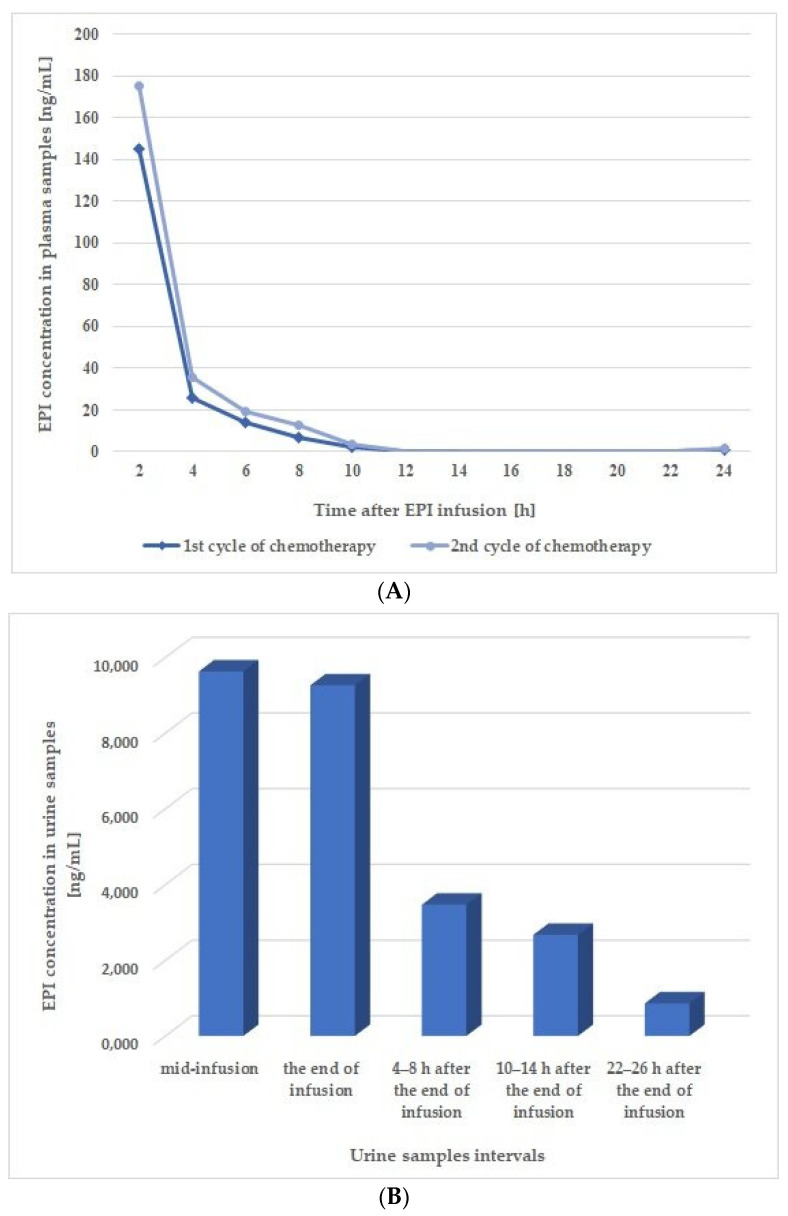
Concentration time profiles of EPI in (**A**) plasma and (**B**) urine samples from a patient receiving this drug at a dose of 150 mg/m^2^ via a 6 h intravenous infusion.

As shown in Figure 3A, the highest concentrations of EPI in the plasma samples, which were collected during two cycles of chemotherapy, were obtained 2 h after the end of the infusion, with values of 175.30 ng/mL and 145.26 ng/mL being recorded for the first and second cycle, respectively. The EPI concentration in the samples taken 4 h after the end of the infusion decreased sharply to values (i.e., 35.92 ng/mL and 25.77 ng/mL for first and second cycle, respectively) several times lower compared to the samples collected 2 h post-infusion, while a mild decrease was observed for the samples collected at all subsequent time-points. Finally, the concentrations of EPI in the plasma samples taken 24 h post-infusion were 1.32 ng/mL and 1.04 ng/mL for the first and second chemotherapy cycles, respectively.

The time–concentration profile of EPI was also obtained for the urine samples collected in the first cycle of chemotherapy. The results presented in Figure 3B prove that EPI can be detected in the patient’s urine halfway through the 6 h infusion (mid-infusion sample). The concentrations of EPI in the mid-infusion samples and those collected immediately after the end of the infusion were 9630.14 ng/mL and 9263.00 ng/mL, respectively, which were the highest values obtained for this matrix. In subsequent samples taken 4–8 h and 10–14 h post-infusion, the concentration EPI was significantly reduced (3473.00 ng/mL and 2675.86 ng/mL, respectively). The EPI concentration was further reduced in the samples taken 24 h after the end of the infusion, although it was still relatively high at 861.57 ng/mL. Representative chromatograms obtained from the analysis of patient plasma and urine samples are shown in Figure S2.

The obtained EPI profiles for the urine and plasma samples were compared to the data from our previous paper, wherein a traditional extraction technique (SPE) was applied for the analysis of EPI in real samples taken from pediatric cancer patient [10]. This comparison was possible due to the fact that both patients had received a 150 mg/m^2^ dose of EPI over a 6 h infusion and samples in both studies were collected at similar time intervals. However, it should be highlighted that the patient in the present study had received EPI in monotherapy, while the patient in the previous study had received it along with vincristine and carboplatin as part of polytherapy [10]. Furthermore, the basic physiological characteristics of the patients, including sex, age, and body surface area (BSA), were also different. Overall, this comparison demonstrated that, despite the similarities in chemotherapy regimens, the concentration time profiles for EPI differ from patient to patient. The plasma samples from the 11-year-old (almost 12 years old) patient in the current study contained lower concentrations of EPI at each measured time point compared to the samples obtained from the patient in our previous study [10]. The significant differences in EPI concentrations observed in the two patients may be related to the therapeutic regimens (monotherapy vs. combination therapy) used in each study or to pharmacokinetic differences. Therefore, it is especially important to monitor both the therapeutic effects and side effects of EPI in individual patients—a recommendation that has been previously asserted for therapies using anticancer agents [28,29,30].

### 3.4. Comparison of the Developed MSPME-Based Method with Previous Reports

Previously reported protocols for the determination of EPI in biological samples have been predominantly based on SPE sampling with commercially available C18 or hydrophilic-lipophilic balance (HLB) columns, although LLE techniques also have been employed in various studies [28,31,32]. However, none of the methods reported in the literature have employed the microextraction technique, which is generally recognized as a simpler, more efficient and eco-friendly approach for sample pretreatment. Moreover, these previously reported methods have not been widely applied for routine drug monitoring in clinical practice; as such, the magnetic-microextraction-based method presented in this work offers an alternative approach to preparing urine and plasma samples that can potentially be implemented in routine laboratory analysis. An important difference between the proposed MSPME method and previously reported SPE or LLE techniques is the former’s significantly reduced organic solvent consumption. In this study, organic solvent was only used for analyte desorption, whereas traditional SPE procedures require large amounts of different organic solvents for column conditioning and for analyte extraction and desorption. Additionally, the optimization of the MSPME procedure (described in Section 3.1) proved that functionalized MNPs can provide high extraction efficiency without any additional sample modification, and that the use of an external magnetic field to separate the sorbents can enable fast analyte isolation. Furthermore, the desorption of EPI with a small amount of ACN ensured the appropriate enrichment factor; thus, it was possible to analyze the sample without additional steps, such as the evaporation and reconstitution of the sample. In SPE, it is often necessary to perform additional extraction steps, such as sample deproteinization to prevent sorbent clogging, SPE column conditioning, or analyte desorption with larger amounts of organic solvents, typically in conjunction with sample evaporation. While sorbent preparation is a key issue in the proposed MSPME method, this study demonstrates that sorbent cores can be quickly prepared using commonly available iron (II) and iron (III) salts and a simple co-precipitation method. Moreover, the wide range of materials available for their functionalization allows the sorbent to be tailored to the analyte more effectively, while the commercially available cartridges used in SPE are mainly limited to C18 or HLB sorbents.

Considering the validation parameters of the developed method, it should be noted that the obtained LOD, LOQ, precision, and accuracy values were comparable to those of traditional extraction techniques. Despite slightly lower extraction efficiencies for EPI (80.40 ± 5.33% for plasma samples and 89.79 ± 2.97% for urine samples) compared to previous protocols, coupling the proposed MSPME method with LC-FL still provides acceptable method sensitivity and the ability to determine even low levels of EPI in real human samples. The results confirming the proposed method’s compliance with ICH and FDA requirements were especially important, as they established its suitability for monitoring EPI in biological samples. Overall, DDAB, which is a cationic surfactant with a double chain alkyl, has not been used previously as a coating for Fe_3_O_4_ cores, and thus, in the development of any method for the analysis of drugs in complex biological matrices.

## 4. Conclusions

In this study, a microextraction protocol based on the coupling of MSPME and LC-FL analysis was developed for the determination of the anticancer drug, EPI, in plasma and urine samples obtained from a pediatric cancer patient. The application of magnetic sorbents facilitated quick separation of the analyte from the sample matrix and significantly simplified the sample-preparation step, while the functionalization of NPs ensured high extraction efficiency for EPI from biological matrices. Importantly, a double-chain surfactant was used as a coating material, which has not been considered previously in the development of MNP-based analytical methods. The volumes of biological material, sorbents, and reagents required for the extraction of EPI enabled the miniaturization of the procedure, establishing the proposed method a promising alternative to previously reported techniques based on traditional SPE and LLE approaches. Additionally, the method optimization enabled insights into the interactions between the analyte and the double-chain-surfactant-based coating. Furthermore, the obtained results proved that the prepared extraction phase allows the isolation of the analyte from biological samples without the need for an initial deproteinization step, which is commonly employed prior to SPE or LLE. The validation results and the data obtained from the analysis of real human samples confirmed the usefulness of the proposed method, which can be also considered more environmentally friendly compared to other approaches and can be implemented in clinical practice for the routine monitoring of EPI concentrations.

## Figures and Tables

**Table 1:** Intra and inter-day precision and accuracy data for the QC samples (n = 6).

Matrices	QCs	Concentration Added [µg/mL]	Precision (RSD (%), n = 6)		Accuracy (%, n = 6)	
			Intra-Day	Inter-Day	Intra-Day	Inter-Day
Plasma	Low	0.1	6.26	7.78	103	95
	Medium	0.5	4.46	3.45	101	102
	High	1.0	2.93	1.97	98	99
Urine	Low	0.5	6.61	8.57	90	90
	Medium	3.0	4.87	5.31	97	104
	High	7.5	3.12	5.02	99	101

## Data Availability

Not applicable.

## Supplementary Materials

The following supporting information can be downloaded at: https://www.mdpi.com/article/10.3390/pharmaceutics15041227/s1. Figure S1: Structure of didodecyldimethylammonium bromide. Figure S2: LC-FL chromatograms obtained for plasma sample collected from the patient 6 h after the end of the infusion and urine samples collected from the patient between 4 and 8 h after the end of the infusion (EPI dose: 150 mg/m^2^, first chemotherapy cycle). Table S1: Stability of EPI in human plasma and urine under various experimental conditions (mean ± SD, n = 3).

## Appendix A. Preparation of Magnetic Sorbents

Fe_3_O_4_-based NPs coated with DDAB were prepared according to the procedure reported in our previous study [17]. Briefly, magnetic Fe_3_O_4_ cores were prepared using a co-precipitation method. The 50 mL of solution consisting of FeCl_3_·6H_2_O and FeSO_4_·7H_2_O salts at ratio of 1:2 (*m*/*m*) and with the addition of concentrated HCl (0.85 mL) were added dropwise to a 1.5 M NaOH solution (100 mL) placed in an ultrasonic bath (80 °C). The obtained NPs were separated from the solution using an external magnetic field, washed several times with an EtOH/H_2_O mixture (80:20, *v*/*v*) and dried in oven (60 °C). After drying, the NP cores were coated with silica. Thus, 0.5 g of bare Fe_3_O_4_ was initially dispersed in 50 mL of 0.1 M HCl and sonicated for 10 min. The NPs were then separated from the 0.1 M HCl solution and added to 50 mL of an EtOH and water (80/20, *v*/*v*) solution along with 1 mL of 25% NH_4_OH and 0.250 µL of TEOS and stirred for 8 h at room temperature. Finally, the coated Fe_3_O_4_ was separated from the solution using a magnet, washed with a mixture of water and EtOH (80/20, *v*/*v*), and dried in an oven at 60 °C. The silica-coated NPs were separated from the solution using an external magnetic field and dried in oven (60 °C). In the final step, the Fe_3_O_4__SiO_2_ was coated with DDAB via the following procedure: 4 mg of surfactant was placed in an Eppendorf tube (2 mL) and dissolved in acetone (0.3 mL); next, 15 mg of NPs were added to the surfactant solution and mixed until the organic solvent had completely evaporated. The obtained Fe_3_O_4__SiO_2__DDAB magnetic sorbents were used in the extraction procedure.
