# Supplementary material for: Magnetic Solid-Phase Microextraction Protocol Based on Didodecyldimethylammonium Bromide-Functionalized Nanoparticles for the Quantification of Epirubicin in Biological Matrices
Source: Pharmaceutics. 2023 Apr 12;15(4):1227. doi: 10.3390/pharmaceutics15041227 (PMC10145736; doi:10.3390/pharmaceutics15041227)
Supplement: Supplementary file 1 [file pharmaceutics-15-01227-s001.zip › pharmaceutics-2314665-supplementary.pdf]

Supplementary Materials

Magnetic Solid-Phase Microextraction Protocol Based on Didodecyldimethylammonium Bromide-Functionalized Nanoparticles for the Quantification of Epirubicin in Biological Matrices

Natalia Treder [1,2], Natalia Szuszczewicz [2], Anna Roszkowska [2], Ilona Olędzka [2], Tomasz Bączek [2], Ewa Bień [3], Małgorzata Anna Krawczyk [3], Alina Plenis [1*]

[1] *Department of Analytical Chemistry, Medical University of Gdansk, Gdansk, Poland*
[2] *Department of Pharmaceutical Chemistry, Medical University of Gdansk, Gdansk, Poland*
[3] *Department of Pediatrics, Hematology and Oncology, Medical University Gdansk, Gdansk, Poland*

* Corresponding author; Phone +48 58 349 10 96; Fax +48 58 349 15 24
E-mail: aplenis@gumed.edu.pl (A. Plenis)

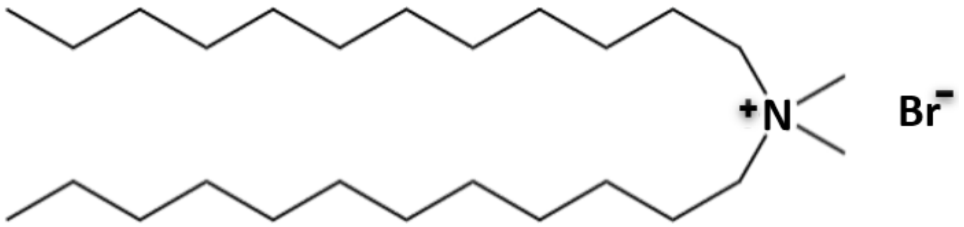

Figure S1. Structure of didodecyldimethylammonium bromide

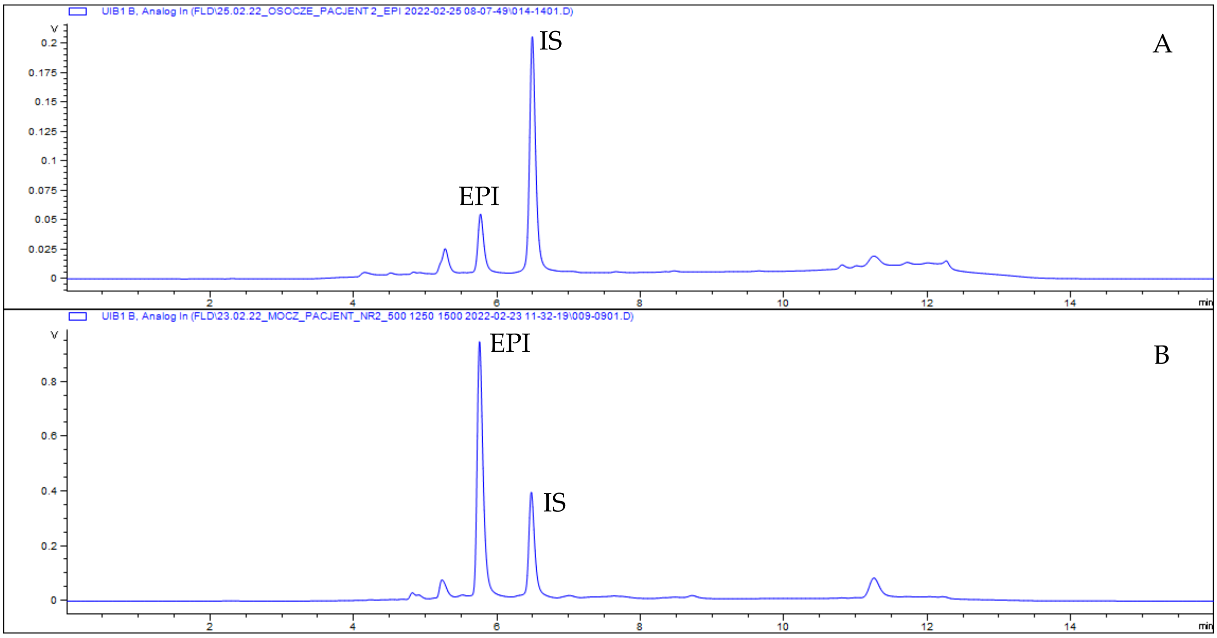

Figure S2. LC-FL chromatograms obtained for patient's plasma sample collected 2h after the end of the infusion (A) and for patient's urine samples collected in the range 4-8 h after the end of the infusion (B) (EPI dose: 150 mg/m^2, first chemotherapy cycle).

Table S1. Stability of EPI in human plasma and urine samples under various experimental conditions (mean ± SD, n = 3).

Storage conditions	Sample	Conc. added (μg/mL)	Precision RSD (%)	Accuracy (%)
Plasma				
Post-preparative storage (4°C, 24 h)	LQC	0.1	10.00	100
	MQC	0.5	11.11	90
	HQC	1.0	9.09	110
Ambient storage (4 h; short-thermal stability)	LQC	0.1	11.11	90
	MQC	0.5	10.00	100
	HQC	1.0	9.09	110
Long-term stability (-80°C, 2 months)	LQC	0.1	14.28	120
	MQC	0.5	14.28	101
	HQC	1.0	12.50	98
Three freeze-thaw cycles stability	LQC	0.1	14.28	120
	MQC	0.5	10.00	100
	HQC	1.0	11.11	110
Urine				
Post-preparative storage (4°C, 24 h)	LQC	0.5	5.77	104
	MQC	3.0	5.81	93
	HQC	7.5	6.93	100
Ambient storage (4 h; short-thermal stability)	LQC	0.5	6.38	94.
	MQC	3.0	6.19	108
	HQC	7.5	5.98	98
Long-term stability (-80°C, 2 months)	LQC	0.5	7.1	112
	MQC	3.0	8.43	110
	HQC	7.5	7.16	99
Three freeze-thaw cycles stability	LQC	0.5	7.2	96
	MQC	3.0	3.8	93
	HQC	7.5	3.4	98
